# New Insights Regarding Hemin Inhibition of the Purified Rat Brain 2-Oxoglutarate Carrier and Relationships with Mitochondrial Dysfunction

**DOI:** 10.3390/jcm11247519

**Published:** 2022-12-19

**Authors:** Daniela Valeria Miniero, Nicola Gambacorta, Anna Spagnoletta, Vincenzo Tragni, Stefano Loizzo, Orazio Nicolotti, Ciro Leonardo Pierri, Annalisa De Palma

**Affiliations:** 1 Department of Biosciences, Biotechnologies and Environment, University “Aldo Moro” of Bari, Via E. Orabona, 4, 70125 Bari, Italy; 2 Department of Pharmacy-Pharmaceutical Sciences, University “Aldo Moro” of Bari, Via E. Orabona, 4, 70125 Bari, Italy; 3 ENEA Italian National Agency for New Technologies, Energy and Sustainable Economic Development, Trisaia Research Centre, S.S. 106 Jonica, Km 419,500, 75026 Rotondella (MT), Italy; 4 Department of Cardiovascular, Endocrine-Metabolic Diseases and Aging, Istituto Superiore di Sanità, Viale Regina Elena, 299, 00161 Roma, Italy

**Keywords:** 2-oxoglutarate carrier, mitochondrial dysfunction, kinetic study, porphyrin derivatives, induced-fit molecular docking, mitochondrial carrier regulatory motif

## Abstract

A kinetic analysis of the transport assays on the purified rat brain 2-oxoglutarate/malate carrier (OGC) was performed starting from our recent results reporting about a competitive inhibitory behavior of hemin, a physiological porphyrin derivative, on the OGC reconstituted in an active form into proteoliposomes. The newly provided transport data and the elaboration of the kinetic equations show evidence that hemin exerts a mechanism of partially competitive inhibition, coupled with the formation of a ternary complex hemin-carrier substrate, when hemin targets the OGC from the matrix face. A possible interpretation of the provided kinetic analysis, which is supported by computational studies, could indicate the existence of a binding region responsible for the inhibition of the OGC and supposedly involved in the regulation of OGC activity. The proposed regulatory binding site is located on OGC mitochondrial matrix loops, where hemin could establish specific interactions with residues involved in the substrate recognition and/or conformational changes responsible for the translocation of mitochondrial carrier substrates. The regulatory binding site would be placed about 6 Å below the substrate binding site of the OGC, facing the mitochondrial matrix, and would allow the simultaneous binding of hemin and 2-oxoglutarate or malate to different regions of the carrier. Overall, the presented experimental and computational analyses help to shed light on the possible existence of the hemin-carrier substrate ternary complex, confirming the ability of the OGC to bind porphyrin derivatives, and in particular hemin, with possible consequences for the mitochondrial redox state mediated by the malate/aspartate shuttle led by the mitochondrial carriers OGC and AGC.

## 1. Introduction

In eukaryotic cells, mitochondria are the site of oxidative metabolism reactions, including the citric acid cycle, the β-oxidation of fatty acids, and oxidative phosphorylation. Although the mitochondrial outer membrane is permeable to small molecules due to the presence of transport proteins called porins [1], the inner membrane is impermeable to ions or small molecules. Therefore, the exchange of many metabolites and cofactors between the cytosol and the mitochondria is necessarily tightly regulated and mostly mediated by a large family of mitochondrial transporters [2,3,4,5,6,7,8,9].

The biosynthesis of heme is an example of a multistep process which requires tight regulation of mitochondria and cytosol to assure the presence of heme precursors and heme itself, crucial both for heme prosthetic group biosynthesis and for the assembly of heme proteins [10,11,12,13]. 

Some studies have proposed that the accumulation of porphyrin derivatives could be mediated by a carrier-transport-like mechanism since porphyrin derivatives are anions, slightly negatively charged at physiological pH, making it difficult for them to cross the inner mitochondrial membrane by passive diffusion [14,15,16].

In heme biosynthesis, the ATP-binding cassette (ABC) transporter sub-family B member 6 (ABCB6) and the mitochondrial peripheral-type benzodiazepine receptor (PBR) have been identified as the best candidates for transporting porphyrins across the outer membrane [16,17,18]. 

Interestingly, many metabolite transporters of the inner mitochondrial membrane, known as mitochondrial carriers (SLC25A family) [2,3,4,5,6,7,8,9], have been involved in several case reports related to the altered concentration of ligands structurally related to porphyrins, as shown in sideroblastic anemia and impaired iron import disease affected patients [15,19]. Furthermore, the mitochondrial 2-oxoglutarate carrier (OGC) and the adenine nucleotide carrier (ANT, also known as ADP/ATP carriers, or AAC) have been investigated for their role in the binding and storage of porphyrin derivatives [20,21,22]. 

Among mitochondrial carriers, the OGC is one of the most studied carriers and plays an important role in the malate/aspartate shuttle [23,24,25]. It has been purified from various sources [26,27], and the transport mechanism has been well characterized [28,29,30,31]. In order to shed light on residues mainly implicated in substrate translocation, a complete cys-scanning mutagenesis was performed and all the recombinant mutant proteins reconstituted into proteoliposomes were investigated through homo-exchange transport assays with radiolabeled substrates [32,33,34,35,36,37,38,39,40]. Furthermore, a broad mitochondrial carrier family analysis quantified the importance of each mitochondrial carrier (MC) protein-residue in the substrate translocation and conformational changes, defining the most important protein regions for mitochondrial carrier function [7,8,40,41].

A new dedicated analysis has recently highlighted the importance of the proposed MC regulatory motif QYKGxxDCxRK, which is shared by most of the MCs in at least one repeat. This motif is located at the level of an MC’s matrix loops and the adjacent short-helices parallel to the membrane plane, between the two portions of the conserved MC signature motif sequence PXD/EXXK/R…..EGXXXXArK/RG [7]. Indeed, the interactions of residues of the sequence motif and/or residues of the regulatory motif on the matrix loops with small molecules have been suggested to participate in the regulation of the activity of the ADP/ATP carrier [7,42]. It was also observed that some of the cited residues might even be responsible for substrate recognition and participate in substrate specificity, as suggested by transport assays following site-directed mutagenesis in the *D. melanogaster* dPCoA carrier and in *S. cerevisiae* Ggc1p [7,41,42,43,44,45,46].

Bioinformatics analysis and the available crystallized structures have allowed researchers to establish that the OGC, as shown for other mitochondrial carriers such as nucleotide carriers [42,43,44], organic acid carriers [45,46,47,48,49,50,51], carnitine, and ornithine carriers, [52,53], has a single binding site for its substrates, i.e., 2-oxoglutarate and malate, which can be alternatively exposed to the intermembrane space or the matrix space, in both the free and substrate-bound states [4,26,54,55,56,57].

Among porphyrin derivatives, hemin is formed from heme during the turnover of old red blood cells, or inappropriately from hemolysis or vascular injury [58]. Its structure is a covalently modified porphyrin ring containing a ferric iron (Fe^3+^) ion with a coordinated chloride ion [59]. Despite being one of the most studied compounds among porphyrins, its role in cell physiology and mitochondrial function is still controversial. On one hand, many papers have reported on the role played by hemin in neuroprotection [60] and in preventing mitochondrial dysfunction [61], and on the effectiveness of the treatment of heme-deficiency-related disorders, such as porphyria [62].

On the other hand, although the mechanism remains elusive, it has been suggested that hemin is involved in promoting colon carcinogenesis due to its implication in the endogenous production of the carcinogenic N-nitroso compounds and the cytotoxic and genotoxic aldehydes by lipid peroxidation [63,64]. Recently, it has also been proposed that hemin, in addition to favoring mitochondrial reactive oxygen species (ROS) production and DNA oxidative damage, can cause a reduction in the activity of complexes I and II of the electron transport chain, with the consequent impairment of mitochondrial membrane potential (MMP) and mitochondrial respiration [63,64,65].

Thus, given the role played by hemin in modulating redox homeostasis and oxidative stress [60,61,62], it is important to gain new molecular details about the interactions of hemin with the OGC, because OGC activity/function is crucial for cell viability due to its involvement in the transfer of reducing equivalents through the shuttle malate/aspartate, led by OGC and the Aspartate/Glutamate carrier (AGC), contributing to the redox balance between cytoplasm and mitochondria [66,67].

From a kinetic point of view, it should be noted that inhibition analyses are performed by building a simple Lineweaver-Burk plot (also called a double reciprocal plot or 1/v vs. 1/S plot), which reflects one of the classical mechanisms, i.e., competitive, uncompetitive, and mixed inhibition [68,69]. Conversely, sometimes a Lineweaver–Burk plot by itself may not be enough to establish the correct mechanism of action of a ligand, and the conclusion, that an inhibitor should bind to the same binding site of the substrate because the double reciprocal plot displays lines intersecting at the Y (ordinate) axis, could be misleading [68,69,70,71].

In the light of these considerations, we employed a set of kinetic equations for describing/analyzing the interaction of hemin with the OGC by plotting the experimental transport data on second-order graphs. Furthermore, we performed new dedicated docking molecular studies on OGC residues belonging to the MC regulatory motifs protruding towards the matrix face of the mitochondrial membrane, to support the kinetic evidence of a partially competitive, instead of a purely competitive mechanism of inhibition showed by hemin. In fact, from our kinetic in vitro assays and molecular modeling analysis, we can propose that hemin interacts with residues of the regulatory binding region located in the loops of the mitochondrial matrix, when reconstituted within proteoliposomes. Thus, it is proposed that at physiological level, hemin can act as a partially competitive inhibitor towards the transport of natural substrates of the OGC, when it interacts with the OGC from the intermembrane space. Conversely, hemin can behave as a mixed inhibitor of OGC antiport activity when it interacts with OGC from the matrix space, causing the formation of an inhibitory “hemin-carrier substrate” ternary complex. On this regard, we can speculate that OGC–hemin interactions can affect the mitochondrial redox state mediated by the shuttle malate/aspartate, which is led by OGC and AGC mitochondrial carriers [66], either when hemin derives from the degradation of heme-dependent cytosolic proteins [22] or when deriving from the degradation of heme-dependent mitochondrial proteins [21], through direct interactions with the OGC matrix loop regulatory site residues.

## 2. Materials and Methods

### 2.1. Chemicals

Hydroxylapatite (Bio-gel HTP) and Amberlite Bio-Beads SM-2 was obtained from Bio-Rad; Matrex Gel Orange was obtained from Amicon (Beverly, MA, USA); Triton X-100, Triton X-114, acrylamide, and N,N′-methylenebisacrylamide were obtained from Serva; egg-yolk phospholipids were obtained from Fluka; hemin, cardiolipin, 1,4-piperazine-diethanesulphonic acid (Pipes), Sodium dodecyl sulfate, (SDS) and asolectin were obtained from Sigma; celite 535 was obtained from Roth; Sephadex G-75 was obtained from Pharmacia; [^14^C] 2-oxoglutarate and [^14^C] malate were purchased from Perkin-Elmer Life Sciences (Waltham, MA, USA). All other chemicals used were of analytical grade. 

### 2.2. Purification of the OGC Carrier

In accordance with the previously described protocols [22,27,72] the 2-oxoglutarate carrier was purified from rat brain mitochondria. Briefly, a solution containing 3% Triton X-100 (*w*/*v*), 20 mM Na_2_SO_4_, 1 mM EDTA, and 10 mM Pipes, with a pH of 7.0, was used to solubilize mitochondria with a final protein concentration of 10 mg/mL. After incubation at 4 °C for 10 min, 4 mg/mL of cardiolipin was supplemented to the mixture and centrifuged at 15,000× *g* for 15 min. Then, the supernatant was applied to cold hydroxylapatite/celite (5:1) columns and eluted in the presence of 3% Triton X-100 (*w*/*v*). The first fraction of 0.6 mL was collected and applied to a cold Matrex Gel Orange column, in accordance with previously reported methods [22,27,72]. The pure OGC was isolated and appeared as a band with an apparent molecular weight of 35 kDa [22]. All of these operations were performed at 4 °C.

### 2.3. Reconstitution of the OGC into Liposomes

The purified OGC was reconstituted into liposomes using a micro-batchwise method in the presence of ion-exchange resin Bio-Beads SM-2, as reported in [72]. The mixture contained 200 μL of the purified OGC, 100 μL of 10% (*w*/*v*) Triton X-114, 80 μL of 10% (*w*/*v*) egg yolk phospholipids in the form of sonicated liposomes, 6 mM 2-oxoglutarate, 200 μL of 10 mg/mL asolectin, and 10 mM Pipes, with a pH of 7.0, in a final volume of 700 μL. This procedure ensures the complete (or predominant) right-side-out direction of insertion of the purified OGC into the proteoliposomes (i.e., as it is in the inner mitochondrial membrane, with the N-/C-termini protruding towards the intermembrane space) [73,74]. The prepared mixture was then transferred into an Eppendorf tube (2 mL) along with 0.4 g Amberlite Bio-Beads SM-2 and, after rotating at 32 rpm, the proteoliposomes were recovered by gentle aspiration. All these operations were performed at room temperature.

### 2.4. Transport Measurements

After the reconstitution process, an exclusion chromatography was performed with a Sephadex G-75 column (0.7 × 15 cm), pre-equilibrated with 50 mM NaCl/10 mM Pipes (pH 7.0) to remove the external substrate, which was not embedded within the proteoliposomes. The fraction eluted from the Sephadex G-75 column (600 μL) was collected and distributed into reaction vessels (100 μL each) for starting transport assays by using the inhibitor stop method [22,23,75]. The transport activity was determined by measuring the transport of labeled substrates from outside to inside (uptake experiments) [27,75]. In these experiments, transport was started by adding the labeled substrates from outside to proteoliposomes containing cold 2-oxoglutarate. This was stopped after 2 min, in the initial linear range of [^14^C]2-oxoglutarate or [^14^C] malate uptake [22], by adding 10 μL of 350 mM pyridoxal 5′-phosphate (PLP), which acted as an inhibitor [75]. External radioactivity was removed by a Sephadex G-75 column (0.6 × 8 cm), and the proteoliposomes were eluted with 1.2 mL of 50 mM NaCl, which were collected in 4 mL of scintillation mixture. In control samples, the inhibitor was added to the labeled substrate at time zero and run in duplicate, with the assay temperature at 25 °C. The transport activity was calculated by subtracting the control value from the experimental values and was expressed as mmol/g protein. The values reported are the means ± SD from three independent experiments.

### 2.5. Protein Quantification

The quantification of the purified OGC carrier was performed through the SDS-polyacrylamide-gel electrophoresis of precipitated proteins in accordance with the methods of Laemmli [76]. Staining was performed via the silver nitrate method [77], and protein concentration was determined via the Lowry method, which was modified for the presence of non-ionic detergents [78]. All the samples used for protein determination were dissolved in 1% (*w*/*v*) SDS.

### 2.6. Computational Studies

The homology model of the OGC and the hemin structure were constructed and optimized as reported elsewhere [22,37]. The exploration of putative regulatory sites within the OGC, which was carried out by the Sitemap package [79], returned four different putative druggable pockets. Among these, only one (see Section 3.4 of the Results section) was further analyzed, being the detected cleft located at the level of the mitochondrial matrix loop residues in correspondence with the proposed MC regulatory sequence motif [8,40,41]. For investigating how hemin could approach the predicted binding region, the induced-fit docking protocol [80] was performed using Glide software [80], setting the OLPS3 force field [81] and the VSGB [82] as the solvation model. Furthermore, the implicit membrane option was also enabled to enhance the reliability of the docking protocol. The implicit membrane is a low-dielectric slab-shaped region, which is treated in the same way as a high-dielectric implicit solvent region. For completeness, the orientation of the AAC carrier within the membrane taken from the OPM database (https://opm.phar.umich.edu/ accessed on 2 December 2022) was used as a template for the generation of the implicit membrane around the OGC carrier, according to the palmitoyl-oleoyl-phosphatidylcholine membrane bilayer size previously estimated for other membrane proteins [44,83]. The enclosing box was centered on the center of mass of OGC residues K62, R170, and Y259. Side-chain conformation predictions were performed on residues within 20 Å from ligand poses, along with Glide SP redocking of each protein–ligand complex structure within 30.0 kcal/mol of the lowest energy pose. 

### 2.7. Statistical Analysis

The values reported in the plots represent the means ± SD from at least three independent experiments. Kinetics parameters were obtained through the reported plots built using the DeltaGraph 5.6 software package (Red Rock Software). Curve fitting and R^2^ calculation were conducted in DeltaGraph 5.6 using a linear regression model for straight lines and a quadratic regression model for the reported curves.

## 3. Results

An accurate kinetic approach and a targeted docking molecular analysis were here employed to provide additional insights regarding the interaction of hemin, a porphyrin endogenous derivative, with the OGC mitochondrial carrier. It has been reported that hemin exerts a competitive inhibition on the 2-oxogutarate uptake in isolated mitochondria [21], and about 50% of inhibition in proteoliposomes reconstituted with the purified OGC (IC_50_ 1.58 ± 0.22 µM for 2-oxoglutarate and IC_50_ 1.66 ± 0.14 µM for malate, respectively) [22]. Therefore, we started with classic kinetic approaches performed on the OGC carrier purified from rat brain mitochondria and reconstituted into proteoliposomes in the presence of outside and inside hemin to investigate hemin’s effects on transport rates in the presence of the physiological substrates, i.e., 2-oxoglutarate and malate. We then processed the results using second order graphs.

### 3.1. Kinetic Analysis of Hemin–OGC Substrate Interactions by Means of a Second-Order Plot

**Figure 1:**
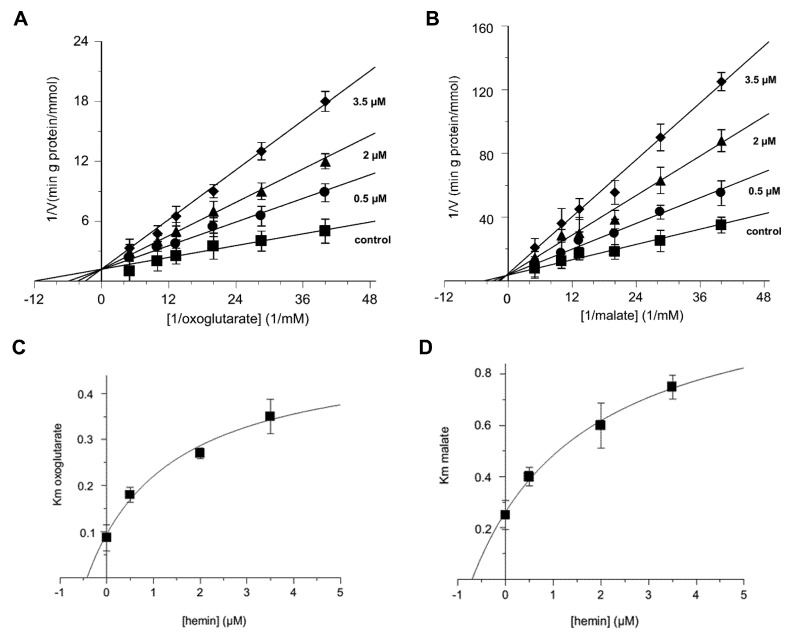
Double reciprocal plots of the 2-oxoglutarate and malate transport activity in presence of external hemin, and second-order graphs. Double reciprocal plots show the dependence of the uptake rate by external concentrations of hemin. The transport rates of [^14^C] 2-oxoglutarate (**A**) and [^14^C] malate (**B**) in proteoliposomes containing 6 mM of 2-oxoglutarate were measured in 2 min in the absence (■) or presence of hemin at 0.5 µM (●), 2 µM (▲), or 3.5 µM (♦). Hemin was added along with [^14^C] 2-oxoglutarate (**A**) and [^14^C] malate (**B**) at concentrations ranging from 0.025 mM to 0.2 mM. The data are displayed as the means ± SD of three different experiments. The R^2^ calculations obtained using a linear regression model for the straight lines of (**A**) were equal to 0.997 in the absence (■) of hemin, and equal to 0.997, 0.995, and 0.952 in presence of hemin at 0.5 µM (●), 2 µM (▲), and 3.5 µM (♦), respectively. The R^2^ calculations obtained using a linear regression model for the straight lines of (**B**) were equal to 0.988 in the absence (■) of hemin, and equal to 0.982, 0.998, and 0.913 in presence of hemin at 0.5 µM (●), 2 µM (▲), and 3.5 µM (♦), respectively. Panel (**C**,**D**) Second-order graphs report the Km’s values obtained from (**A**) (R^2^ = 0.962) and (**B**) (R^2^ = 0.990) versus hemin concentrations.

The double reciprocal graph of Figure 1A,B reports lines crossing the ordinate axis at a point, indicating that hemin behaved like a competitive inhibitor. However, only from this type of diagram, the true mechanism of the inhibition, i.e., whether it was pure or partial competitive, cannot be established [68,69,70,71]. A more accurate representation of the data from Figure 1A,B can be obtained using second-order plots, in which Km’s values are reported with regard to hemin concentrations. These plots are better suited to saturation curves typical of a partial competitive inhibition (also known as hyperbolic inhibition) and not to straight lines, as would be expected from a pure competitive inhibition (Figure 1C,D) [68,69,70,71]. In fact, the quadratic regression analysis produced more appreciable coefficients, i.e., R^2^ = 0.990 for Figure 1D, than those obtained by using the linear regression (R^2^ = 0.954). Furthermore, the non-linear fitting allows us to evaluate the K_E_, i.e., the dissociation constant of the hemin from the possible ternary malate-carrier-hemin complex facing the outside of the proteoliposomes, which is equal to 2.8 ± 0.24 μM and graphically corresponds to the vertical asymptote of the hyperbola [E] = −K_E_

### 3.2. Effects of External Hemin on Malate Uptake in the Reconstituted OGC

To better understand the results reported above, the rate of the labeled [^14^C] malate uptake was investigated as a function of different external hemin concentrations in exchange with the internal 2-oxoglutarate in proteoliposomes reconstituted with the purified OGC. This was in line with the main physiological role of the OGC carrier in the brain, where it is maximally efficient in catalyzing the exchange between the external malate and internal 2-oxoglutarate [27] due its role in the malate/aspartate shuttle, which allows the transfer of reducing equivalents from cytosolic NADH to mitochondrial NAD^+^, especially during the glycolytic process.

**Figure 2:**
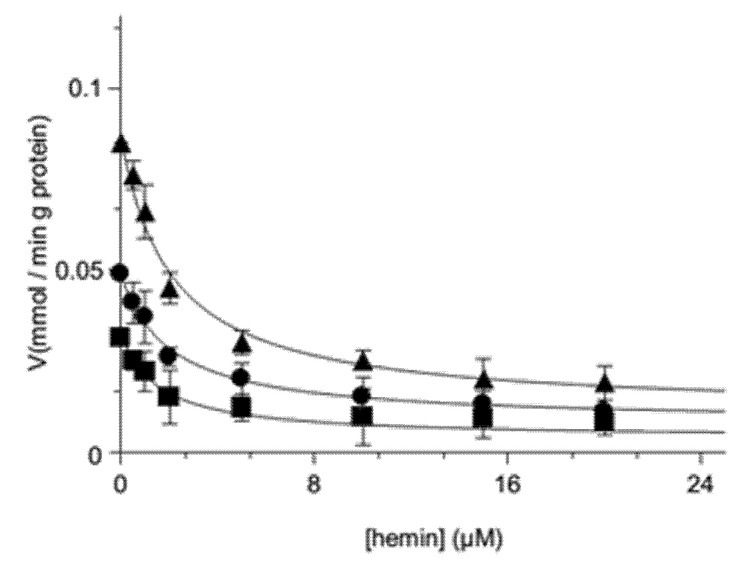
Inhibition of the transport activity of [^14^C] malate on the reconstituted OGC by external hemin. The transport rate of [^14^C] malate was measured in 2 min in the absence or in the presence of increasing concentrations of external hemin (0.5–20 µM). [^14^C] Malate was added along with hemin at a concentration of 0.025 mM (■, R^2^ = 0.978), 0.05 mM (●, R^2^ = 0.993), or 0.1 mM (▲, R^2^ = 0.987) to proteoliposomes containing 6 mM of 2-oxoglutarate. The values presented are the means ± SD of three independent experiments.

Thus, different concentrations of [^14^C] malate were used, and the results of Figure 2 clearly show a decrease in transport rates for different asymptotic curves.

For purely competitive inhibitors at infinite (saturating) concentrations, the rate of substrate transport is reduced to zero, whatever the substrate concentrations were, implying that in the plot, the family of curves had the abscissa as a common asymptote. In various performed experiments, the tentative fitting of the data according to this interpretation was unsuccessful, as shown by the curves parallel to the abscissa axis at increasing concentrations of hemin (Figure 2). Such a behavior is typical of a partially competitive inhibition mechanism, which would be coherent with the formation of a ternary substrate-carrier–hemin complex.

**Figure 3:**
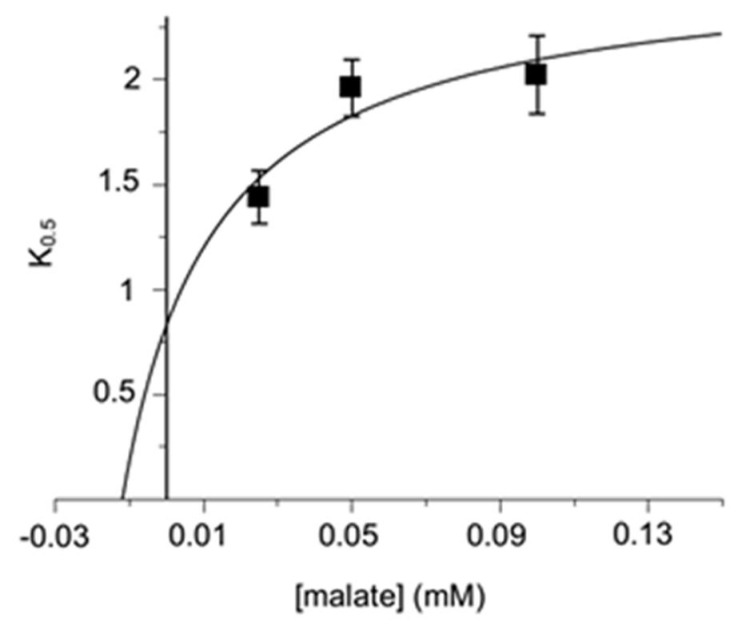
Second-order plot of the data from Figure 2. The dependence of K_0.5_ was obtained from Figure 2 on the external labeled malate. The R^2^ calculation obtained using a quadratic regression model was equal to 0.862, better than the one obtained by using a linear regression model (0.665).

Furthermore, when plotting the K_0.5_ values extrapolated from each curve of the graph of Figure 2 versus malate concentrations, the points showed a non-linear trend, as they should have been in a pure competitive inhibition, but align better with a saturation curve, as reported in Figure 3. The implication of this result was not trivial because a greater number of equilibria must be taken into account in order to define an equation that satisfies the trend of the curve of Figure 3, which is, again, coherent with the formation of a ternary substrate-carrier-inhibitor complex. On this concern new parameters in the kinetic equations have been considered (see Section 4) [68,69,70,71]. 

### 3.3. Effects of Internal Hemin on Malate Uptake in the Reconstituted OGC 

**Figure 4:**
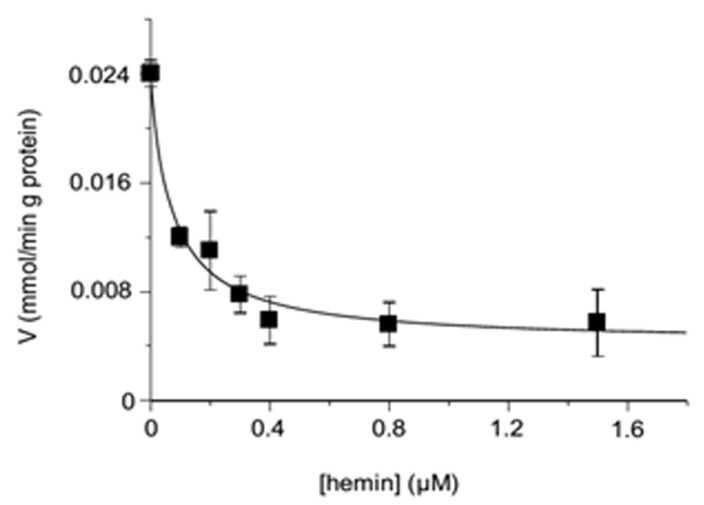
Inhibition of the transport rate of the [^14^C] malate in the reconstituted OGC carrier by internal hemin. The transport rate of [^14^C] malate uptake at a concentration of 0.025 mM was measured in 2 min in proteoliposomes containing 6 mM of 2-oxoglutarate, in the presence of internal increasing concentrations of hemin (0–1.5 μM). The values displayed are the means ± SD of three independent experiments (R^2^ = 0.986).

Just as the inhibition by hemin of transport rates in proteoliposomes reconstituted with the OGC were assessed when hemin was added externally along with the labeled malate, in another set of experiments, the effects of hemin reconstituted internally within proteoliposomes were analyzed. The results reported in Figure 4 provide clear evidence that hemin showed a powerful inhibitory effect on the malate substrate influx in the order of nanomolar concentrations (IC_50_ = 0.082 ± 0.007 μM), indicating an effective and specific interaction with the OGC carrier. 

**Figure 5:**
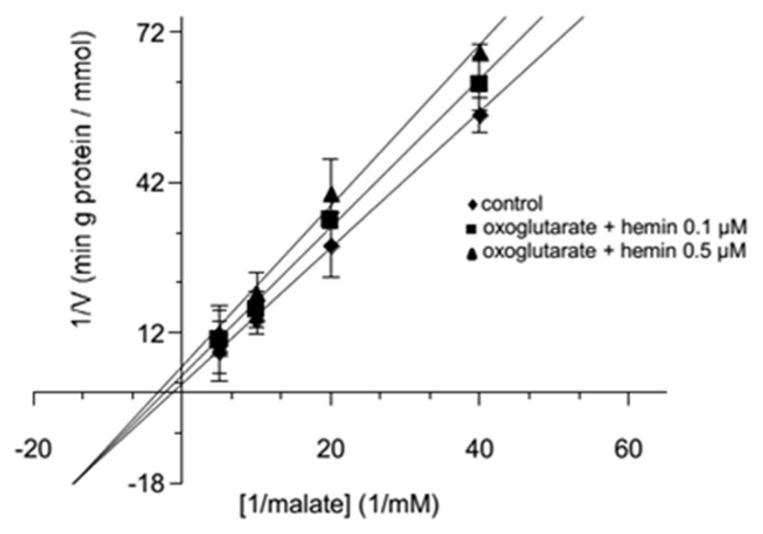
Lineweaver-Burk plot of the [^14^C] malate uptake in the reconstituted OGC carrier in the presence of internal hemin. The transport rate of [^14^C] malate uptake in proteoliposomes containing 6 mM 2-oxoglutarate (♦, R^2^ = 0.997), 6 mM 2-oxoglutarate plus 0.1 μM (■, R^2^ = 0.993) and 0.5 μM (●, R^2^ = 0.994) of hemin was measured in 2 min. [^14^C] Malate was added at a concentrations of 0.025 mM, 0.05 mM, 0.1 mM, or 0.2 mM. Three independent experiments were performed and the mean of values ± SD are reported.

Finally, when the rate of [^14^C] malate influx in proteoliposomes containing hemin was evaluated as a function of the malate concentrations, the double reciprocal plot showed straight lines which met in the third quadrant of the diagram (Figure 5) with a decrease in both the V_M_ and the K_M_ values [68,69,70,71].

### 3.4. Computational Analysis for Investigating the Interactions between OGC Residues and Hemin at the Level of the Proposed MC Regulatory Sequence Motif

**Figure 6:**
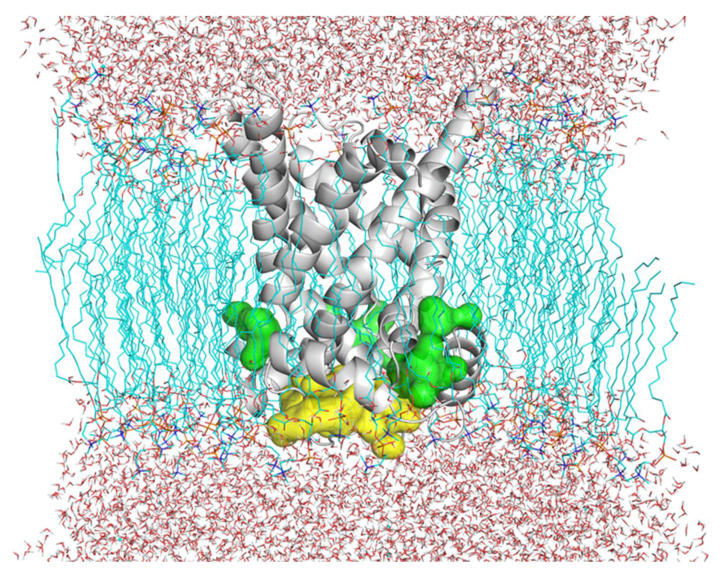
The OGC carrier structure within the inner mitochondrial membrane (displayed as cyan sticks) is represented by the gray cartoon. Water molecules above and below the represented membrane are also displayed as white and red sticks. The predicted binding sites facing the membrane, resulting from the Sitemap analysis, are represented by green surf. The predicted binding site, facing the mitochondrial matrix, is represented by yellow surf. The highlighted binding sites facing the membrane were excluded from the following docking analyses because they were not accessible for the substrate.

The presented computational studies were conceived aiming at investigating the existence of a possible further hemin binding region on the OGC surface, beyond the substrate binding area. Being the OGC located within the mitochondrial inner membrane, three out of the four Sitemap-predicted binding sites (Figure 6) were excluded from the following analyses due to their localization at the protein–membrane interface.

**Figure 7:**
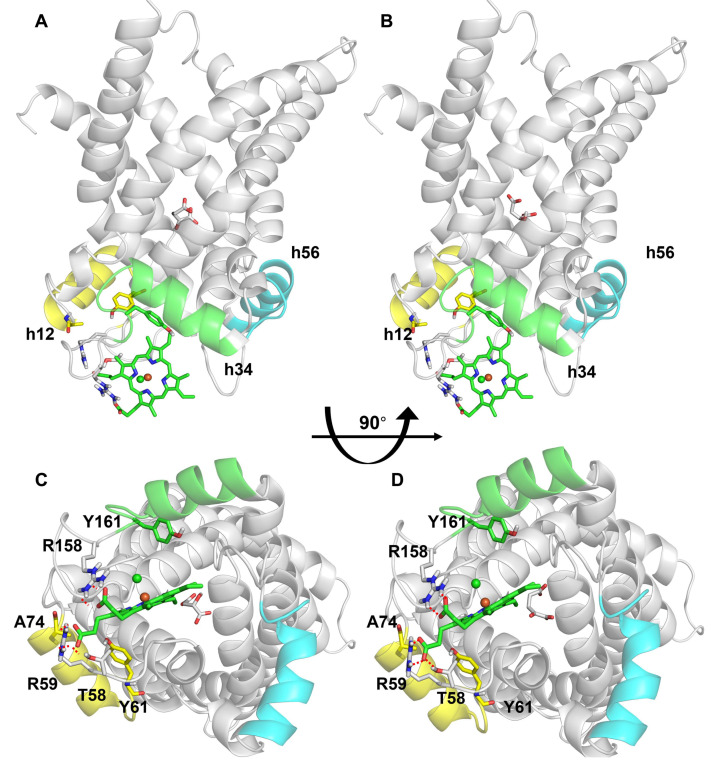
Induced-fit docking results. Panels (**A**,**B**) report the side view of ternary complexes of OGC–malate–hemin and OGC–2-oxoglutarate-hemin, respectively. The matrix short helices h12, h34, and h56 are colored in yellow, green, and cyan, respectively, and are labeled. Panels (**C**,**D**) show the bottom views of OGC–malate–hemin and OGC–2-oxoglutarate-hemin ternary complexes, respectively. The best hemin-docked pose produced through IF docking analysis is depicted using green sticks. The red dotted lines indicate hydrogen bonds between hemin and the closest OGC residues among those explored in the docking analyses.

Indeed, the presence of the phospholipids means that the OGC surface is not freely accessible to hemin, preventing hemin–OGC binding interactions. Notably, the last remaining predicted binding site, facing the mitochondrial matrix, was in correspondence with MC matrix loops, at the level of a MC protein region known for being involved in the regulation of MC activity [7]. Thus, this predicted binding site was chosen for the following docking analysis (Figure 6). Molecular docking simulations were performed to suggest and evaluate a reasonable binding of hemin to the matrix loop regions of the OGC carrier, in presence of the substrate in the similarly located MC substrate binding region, thus endorsing the hypothesis of the ternary complex formation. In particular, as shown in Figure 7, hemin can bind to the OGC carrier from the mitochondrial matrix by establishing a network of polar interactions with the side-chains of T58, R59, and R158, and other close hydrophilic/positively charged residues located in the matrix loops and involved in the correct behavior of the carrier, as demonstrated by site-directed cys-scanning mutagenesis assays [8,35]. Interestingly, the hemin porphyrin ring was also found within 6 Å from residues A74 and Y61 (h12 short matrix helix) and within 8 Å from Y161 (h34 short matrix helix), which may stabilize hemin orientation through aromatic interactions along conformational changes, making closer short helices parallel to the membrane plane [7,84]. To ensure completeness, the docking score value calculated for the presented pose (Figure 7) was equal to –5.42 kcal/mol. 

## 4. Discussion

The 2-oxoglutarate carrier of rat brain mitochondria has been the object of detailed kinetic studies, which were coherent with the single binding center gated pore mechanism [27,44,54,55,84,85]. According to this mechanism, as demonstrated for the ADP/ATP carrier [43,44,48,79], tricarboxylate carrier [50,56], and other MCs sharing a similar substrate translocation mechanism [8,27,45,51,52,86,87], the OGC carrier has a single binding site which can be exposed alternately towards the intermembrane space or the matrix space. The rearrangement of the transmembrane helices, funneling the substrate translocation pathway, gives rise to the formation of the carrier–substrate complex in the c-conformation or in the m-conformation [7,8,27,39,51,52].

However, the 2-oxoglutarate carrier is maximally efficient to catalyze the exchange between the external malate and internal 2-oxoglutarate, in line with its main physiological role, especially in the brain, due to its involvement in transferring reducing equivalents through the malate/aspartate shuttle, ensuring a correct redox balance between mitochondria and the cytoplasm [66,67].

Notably, the effects of some porphyrin derivatives on the activity of mitochondrial carriers have been reported firstly by Kabe et al. [21]. The authors showed that porphyrin accumulation in mitochondria is mediated by the OGC, and that porphyrins can competitively inhibit 2-oxoglutarate uptake into mitochondria [21]. More recently, the effects of hemin on OGC activity were investigated by reconstituting the purified OGC into proteoliposomes, showing also that hemin was able to inhibit the OGC competitively, although in Dixon plots, the results hardly fit to straight lines as one would expect from a purely competitive inhibition [22].

Here, we investigated in a more accurate way the inhibition exerted by hemin, gaining new clues about a partially competitive mechanism of inhibition. It was observed that hemin could bind to the OGC from the cytosolic face, as physiologically may occur because of the degradation of the pool of porphyrin/heme derivatives out from mitochondria [22,88]. 

**Figure 8:**
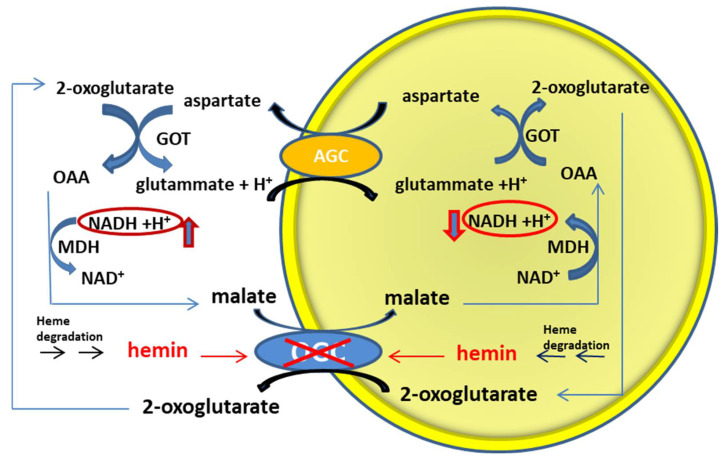
Malate/aspartate shuttle and the effects of hemin. The scheme represents the mitochondrial inner membrane with the components of the malate/aspartate shuttle, i.e., the OGC carrier (coded by the SLC25A11 gene) and the AGC carrier (coded by the SLC25A12/SLC25A13 genes). The presence of hemin, both inside and outside the mitochondria, derived from the degradation of porphyrin/heme pools, inhibits the OGC carrier, causing the consequent impairment of the transfer of reducing equivalents between the cytoplasm and the mitochondrial matrix.

Furthermore, it has been here shown that hemin may behave as a mixed inhibitor when reconstituted within proteoliposomes, implicating that it would bind the OGC from the matrix face to form a ternary hemin–carrier–substrate complex, as a physiological degradation of the pool of porphyrin/heme derivatives active as cofactors of protein complexes located within the inner mitochondrial membrane or in the mitochondrial matrix [88]. As consequence of these interactions, the inhibition of the OGC carrier by hemin would involve an imbalance of the reducing equivalents inside and outside the mitochondria with the alteration of the cellular oxidative state (Figure 8). 

The molecular docking analysis here presented has allowed us to demonstrate a possible additional way of hemin binding to the OGC, thanks to the formation of stable interactions between hemin and residues of the regulatory motif located at the level of the matrix short helices h12 and h34. It is proposed that these interactions could be involved in the regulation of OGC activity, and thus of the malate/aspartate shuttle, which is responsible for the redox balance between mitochondria and cytoplasm. Furthermore, aromatic residues of the regulatory motif, already known to play a role in redox reactions and the regulation of several MCs [89,90], can also be implicated in the electron transfer processes supported by the presence of hemin, which can work as an electron carrier [91]. Moreover, residues of the hemin binding region proposed to participate in these electron transfer processes are in the 10Å range of distances from hemin, a distance which is also largely compatible with electron tunneling processes [83,92], which can be important for redox reactions involved in the assembly of protein super-complexes [93,94,95,96,97].

**Figure 9:**
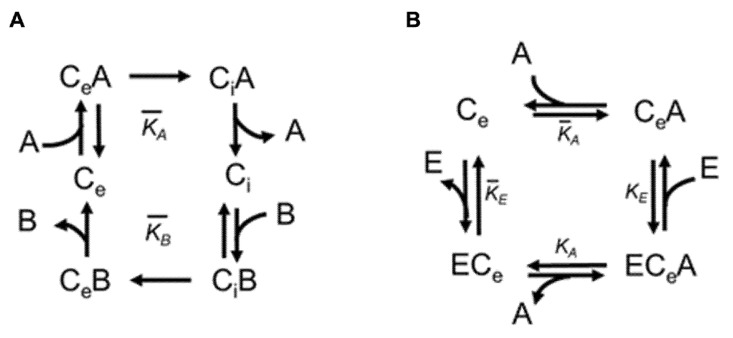
Schematic catalytic cycle. Panel (**A**) shows a simplified schematic representation of the catalytic transport cycle of the carrier (C) with the two substrates malate (A) and 2-oxoglutarate (B) with the corresponding equilibrium constants. Panel (**B**) shows the catalytic reactions with the equilibrium constants in the presence of hemin (E) (preloaded within proteoliposomes), with the formation of the ternary complex. “Ce” and “Ci” indicate the two possible conformations that the carrier undergoes along the transport cycle, i.e., open towards the intermembrane space, “Ce”, or towards the mitochondrial matrix, “Ci.”

From a kinetic point of view, an elaboration of the kinetic equations describing the malate transport rate mediated by the OGC has been performed based on the described experimental results, to evaluate whether they fit with the classic Michaelis-Menten kinetics. Thus, the initial transport rate (v) of external substrate “A” [^14^C] malate was experimentally measured in the purified and reconstituted OGC “C“ containing internal 2-oxoglutarate “B”, in the presence of external hemin “E” (Figure 1B and Figure 9).

An equation can be derived by considering the dissociation equilibrium constants of the expected binary complexes that can be formed between the carrier and the two substrates (Figure 9A), and of the two possible ternary complexes that can be formed with the radiolabeled substrate (in this case [^14^C] malate) and hemin characterizing the fast stages of the transport process (Figure 9B):

$${\bar{K}}_{E}=\frac{\left[E\right]\cdot {\left[C\right]}_{e}}{{\left[EC\right]}_{e}}\;$$

$${\bar{K}}_{A}=\frac{\left[A\right]\cdot {\left[C\right]}_{e}}{{\left[CA\right]}_{e}}\;\;\;\;\;\;{\bar{K}}_{B}=\frac{\left[B\right]\cdot {\left[C\right]}_{i}}{{\left[CB\right]}_{i}}$$

$$K_{E}=\frac{\left[E\right]\cdot {\left[CA\right]}_{e}}{{\left[ECA\right]}_{e}}\;\;\;\;\;\;K_{A}=\frac{\left[A\right]\cdot {\left[EC\right]}_{e}}{{\left[ECA\right]}_{e}}$$

Notably, as shown below, the equilibrium constant relating to the ternary ECB complex was not reported because the uptake the [^14^C] malate (A), and not the 2-oxoglutarate (B), was monitored.

$$EC_{i\;}B\;\begin{array}{c} K_{B}\\ ⇆ \end{array}\;EC_{i\;}+B$$

For the equilibrium constants, the Haldane relation was valid, and the term “f” represented a ratio of equilibrium constants [98].

$$f=\frac{{\bar{K}}_{E}}{K_{E}}=\frac{{\bar{K}}_{A}}{K_{A}}\;$$

$$K_{E\;}=\frac{1}{f}\bar{K}{}_{E}\;\;\;\mathrm{and}\;\;\;K_{A}=\frac{1}{f}{\bar{K}}_{A}$$

The kinetic constants of the rearrangement stages of the flexible OGC conformations were the slow stages which determined the rate of the transport process:

$$\;[ECA{]}_{e}\;\begin{array}{c} k_{4}\\ ⇆\\ k_{-4} \end{array}\;[ECA{]}_{i\;}\;[CA{]}_{e}\;\begin{array}{c} k_{4}\\ ⇆\\ k_{-4} \end{array}\;[CA{]}_{i}\;[CB{]}_{e}\;\begin{array}{c} k_{2}\\ ⇆\\ k_{-2} \end{array}\;[CB{]}_{i}\;[C{]}_{e}\;\begin{array}{c} k_{3}\\ ⇆\\ k_{-3} \end{array}\;[C{]}_{i}$$

The carrier-malate and hemin-carrier-malate complexes (which we monitored by following the uptake of the radiolabeled substrate) would carry out the rearrangements inside the liposomal membrane, through which the substrate can be transferred in the presence and/or absence of the hemin.

It is observed that the rate of the transport depended on the rate of the slow stages, i.e., it was given by:

$$v=k_{4}{\left[ECA\right]}_{e}+k_{4}{\left[CA\right]}_{e}\;$$

Overall, in the steady state in which the transport rate remained constant, the concentrations of the different forms of the carrier also remained stationary, depending on the various listed constants, as well as on the concentration of the substrate and hemin. Therefore, using the above relations, [CA]_e_ and [ECA]_e_ can be expressed as functions of the concentration of the substrate, the equilibrium constants, and the kinetic constants, deriving the following equation:

(1) $$V=k_{4}C_{T}\frac{F_{i}}{F_{i}+k_{4}G_{i}}\cdot \frac{\left(\left[E\right]+K_{E}\right)\cdot \left[A\right]}{\left[E\right]\cdot \left[A\right]+K_{E}\cdot \left[A\right]+\frac{F_{i}}{F_{i}+k_{4}G_{i}}\cdot K_{A}\left[E\right]+\frac{F_{i}+k_{3}G_{i}}{F_{i}+k_{4}G_{i}}\cdot f\cdot K_{A}K_{E}}$$

in which total concentrations, C_T_, were equal to the contribution of all species involved in the transport process:

$$C_{T}=[ECA{]}_{e}+[EC{]}_{e}+[CA{]}_{e}+[C{]}_{e}+[ECB{]}_{i}+[EC{]}_{i}+[CB{]}_{i}+[C{]}_{i}$$

For further simplification, F*i* and G*i* were also introduced as functions of the concentration of B within the proteoliposomes and were therefore constant as long as the concentration of B was kept constant:

$$F_{i}=k_{-2}\left[B\right]+k_{-3}{\bar{K}}_{B}\;\;\;\;\;\;\;and\;\;\;\;\;\;\;\;\;G_{i}=\left[B\right]+{\bar{K}}_{B}$$

Thus, it was necessary to verify that the experimental results fit the general theoretical equation and those derived from them to demonstrate the hypothesis that hemin was a partially competitive inhibitor of the transport of the malate in the OGC carrier reconstituted in proteoliposomes. It was also necessary to try to understand whether hemin binds the carrier from the same face of the entering radiolabeled substrate (as described in [22]), from the opposite side, or from both sides (depending on the mitochondrial compartment, i.e., whether the intermembrane space or matrix space was supplying hemin) to form a ternary substrate-carrier-inhibitor complex.

Starting from the general Equation (1), the transport rate, V, can be expressed (1) as a function of [A], the external concentration of the malate, maintaining [E], the external concentration of hemin, as a constant, and (2) the transport rate, V, can be expressed as a function of [E] when [A] is kept constant. 

In the first case, for each concentration of E, the equation describing the Michaelis-Menten trend was obtained and, therefore, the graph of double reciprocal met on the ordinate axis. In other words, we obtained hyperbola whose equation was:

$$V=V_{M}\cdot \frac{\left[A\right]}{\left[A\right]+K_{M}}$$

and the related double reciprocal was:

(2) $$\frac{1}{V}=\frac{1}{V_{M}}+\frac{K_{M}}{V_{M}}\cdot \frac{1}{\left[A\right]}$$

where V_M_ and K_M_ were respectively equal to:

$$V_{M}=k_{4}C_{T}\frac{F_{i}}{F_{i}+k_{4}G_{i}}=V^{\prime }$$

$$K_{M}=\frac{\left[E\right]+\frac{F_{i}+k_{3}G_{i}}{F_{i}}\cdot f\cdot K_{E}}{\left[E\right]+K_{E}}\cdot \frac{F_{i}}{F_{i}+k_{4}G_{i}}\cdot K_{A}=\frac{\left[E\right]+K^{{\bar{\prime }}_{E}}}{\left[E\right]+K_{E}{}_{A}^{\prime }}$$

To simplify the expression, the equilibrium constants $K_{A}^{\prime }$ and ${\bar{K}}_{E}^{\prime }$ were also introduced:

$${K^{\prime }}_{A}=\frac{F_{i}}{F_{i}+k_{4}G_{i}}\cdot K_{A}\;\;\;\mathrm{and}\;\;\;{{\bar{K}}^{\prime }}_{E}=\frac{F_{i}+k_{3}G_{i}}{F_{i}}\cdot f\cdot K_{E}$$

It should be noted that the experimental data reported in the double reciprocal plot of Figure 1B fit well with the corresponding Equation (2), in which the V_M_ was independent from [E], while the K_M_ was not (Figure 1D). 

On the other hand, in the second case in which the rate (V) was expressed as a function of the concentration of E, where the concentration of A was kept constant, the equation of rate V was expressed as a function of [E], which described a decreasing hyperbolic graph represented by the equation below.

(3) $$V=V_{\sigma }\cdot \frac{\left[E\right]+K_{E}}{\left[E\right]+K_{0.5}}$$

where

(4) $$V_{\sigma }=V^{\prime }\cdot \frac{\left[A\right]}{\left[A\right]+{K^{\prime }}_{A}}\;\mathrm{and}\;K_{0.5}=\frac{\left[A\right]+\frac{F_{i}+k_{3}G_{i}}{F_{i}}\cdot f\cdot {K^{\prime }}_{A}}{\left[A\right]+{K^{\prime }}_{A}}\cdot K_{E}$$

Vσ, the rate in the presence of saturating hemin concentrations, represented the horizontal asymptote that, as with K_0.5,_ varied in function of the concentration of A. Additionally, V_0_, varied in function of the concentration of A and was defined by the expression:

$$V_{0}=V_{\sigma }\cdot \frac{K_{E}}{K_{0.5}}$$

Vσ was equal to 0 only when A was 0, i.e., in the absence of substrate, and this was important to discriminate between the two types of competitive inhibition. In fact, if it was a purely competitive inhibitor, the related equation would more similar to:

(5) $$v=V_{0}\cdot \frac{K_{0.5}}{K_{0.5}+\left[E\right]}$$

In this case, while the parameters V_0_ and K_0.5_ were always function of the external concentration of malate (A), the asymptote represented the abscissa axis at any concentration of the malate.

As shown in Figure 2, where the transport rate of [^14^C] malate in proteoliposomes was measured as a function of increasing concentration of hemin in the presence of three different labeled malate concentrations (0.025–0.1 mM), our experimental data fit Equation (3) rather than (5). In fact, three decreasing hyperbolic curves were obtained (Figure 2), in which the asymptotes Vσ were clearly distinct from the abscissa axis in agreement with the hypothesis of partially competitive inhibition.

Finally, by plotting the K_0.5_ values extrapolated from each curve of the graph of Figure 2 as a function of the malate concentrations, the points tended to be not aligned, but were better suited to the expected increasing hyperbolic Equation (4), as shown in Figure 3.

Of course, to verify the effect of hemin inside the proteoliposomes, we monitored the uptake of the external [^14^C] malate in the presence of increasing inner concentrations of hemin. It appears evident that the transport rate was decreased by the presence of hemin within proteoliposomes, as observed in Figure 4. These results aligned perfectly with the hypothesis of a ternary complex “hemin-carrier-substrate”, as also supported by the experimental data of Figure 5, in which three straight lines tended to meet in the third quadrant, as expected for a mixed inhibitor (according to [68,69,70,71]). Thus, it is expected that the hemin binds to a regulatory site, immediately before or after the substrate binding. In addition, it is also retained that this regulatory site is located in a different region with respect to the accessible substrate binding site, suggesting the formation of the proposed ternary “substrate-carrier-hemin” complex.

However, although the existence of the ternary complex “hemin-carrier-substrate” is suggested by kinetics analysis, only a crystallized structure might provide certain atomic coordinates of such a ternary complex. Conversely, in order to shed light on a possible binding site of hemin within a ternary hemin-carrier–substrate complex, a computational docking-based analysis was performed. The performed analysis suggested that the best candidate-predicted hemin binding site was located close to the proposed regulatory sequence motif residues facing the mitochondrial matrix. The exploration of the predicted hemin binding site returned a reliable set of polar interactions between hemin with the basic residues R58, R59, and R158, the latter of which is also important for the transport activity [35]. Notably, the highlighted residues are in the proximity of the proposed regulatory sequence motif [8,40,41] on the short helices h12 (60-EYKTSFHALISILRA-74) and h34 (160_GYKNVFNALFRIVQE-174), parallel to the membrane plane.

To provide more detail, the aromatic residues Y61 and Y161 are between 5Å and 8Å far from hemin in the generated 3D model, which was obtained by using the BtAAC1-carboxyatractyloside protein-inhibitor complex as a protein template [5]. Although the distance between hemin and the cited aromatic residues of the MC regulatory motif is apparently great in the 3D comparative model [7], the deep conformational changes necessary for substrate translocation and the following release of the substrate towards the intermembrane space may cause the reorientation of short helices parallel to the membrane plane, taking them closer than what observed in the 3D model. In such conditions, it is suggested that aromatic residues of OGC short helices parallel to the membrane plane may form an aromatic pocket for interactions with hemin pyrrole rings.

## 5. Conclusions

Our experimental data, performed on an isolated system represented by the proteoliposomes, confirmed the interaction of the purified rat brain mitochondrial OGC carrier with hemin, a physiological product of heme degradation. Hemin acts as a partially competitive inhibitor towards the transport of natural substrates of the OGC when added externally, and as a mixed inhibitor when reconstituted within proteoliposomes. 

Concerning the question of the right-side-out orientation of the OGC, it is assumed that the OGC is predominantly inserted in the right-side-out orientation when reconstituted in proteoliposomes (i.e., as it is in the inner mitochondrial membrane, with the N-/C-termini protruding towards the intermembrane space) [73,74]. Therefore, we could suggest that hemin coming from the matrix space can bind in vivo to some OGC matrix loop residues implicated in the substrate recognition and/or conformational changes responsible for the translocation of the substrates, giving rise to a ternary complex. Although additional studies involving biophysical and structural approaches could provide further contributions in understanding the interactions of the OGC with porphyrin derivatives, our previous analysis [22] and the new results here presented, allow us to speculate that the OGC is targeted/impaired by hemin produced along the degradation of porphyrin derivatives coming from the cytoplasm or from the mitochondrial matrix under oxidative stress conditions [98]. The proposed interactions between porphyrin derivatives and the OGC might regulate the malate/aspartate shuttle contributing to the maintenance or the impairment of the redox balance between mitochondria and cytoplasm [66,91,92,94,95,96], and consequently it may also play a role in the regulation of mitochondrial apoptosis [98,99,100,101,102,103,104,105].

## Data Availability

The data supporting the findings of this study are available within the article. Raw data that support the findings of this study are available from the corresponding authors, upon request.
